# Supplementary material for: A toolbox of astrocyte-specific, serotype-independent adeno-associated viral vectors using microRNA targeting sequences
Source: Nat Commun. 2023 Nov 16;14:7426. doi: 10.1038/s41467-023-42746-w (PMC10654773; doi:10.1038/s41467-023-42746-w)
Supplement: Supplementary file 1 — Supplementary Information [file 41467_2023_42746_MOESM1_ESM.pdf]

Supplementary Figure 1

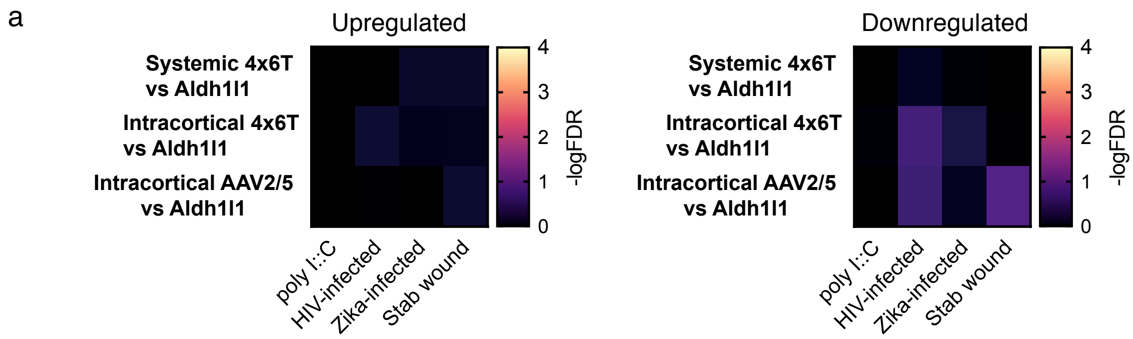

Supplementary Figure 1. GSEA comparison of astrocytic gene changes induced by AAV transduction vs viral infection or stab wound. a, Up- and down-regulated gene sets associated with astrocytic infection by HIV or Zika virus, viral mimetic poly I::C, or stab wound injury were compared with gene changes in AAV-transduced astrocytes (vs Aldh1l1-CreERT2 transcriptomes). None of these gene sets were enriched (FDR < 0.05; -logFDR >1.30103), suggesting that the gene changes induced by AAV transduction are not reminiscent of the gene changes astrocytes undergo in these pathologic conditions.
