# Supplementary material for: A toolbox of astrocyte-specific, serotype-independent adeno-associated viral vectors using microRNA targeting sequences
Source: Nat Commun. 2023 Nov 16;14:7426. doi: 10.1038/s41467-023-42746-w (PMC10654773; doi:10.1038/s41467-023-42746-w)
Supplement: Supplementary file 3 — Description of Additional Supplementary Files [file 41467_2023_42746_MOESM3_ESM.docx]

**Description of Additional Supplementary Files**

**Supplementary Data 1**: RNAseq gene lists: IP samples, FDR < 0.05

**Supplementary Data 2**: GSEA gene sets in viral cohorts, FDR < 0.05

**Supplementary Data 3**: Potential miR target genes, FDR < 0.05
