# Supplementary material for: A toolbox of astrocyte-specific, serotype-independent adeno-associated viral vectors using microRNA targeting sequences
Source: Nat Commun. 2023 Nov 16;14:7426. doi: 10.1038/s41467-023-42746-w (PMC10654773; doi:10.1038/s41467-023-42746-w)
Supplement: Supplementary file 7 — Reporting Summary [file 41467_2023_42746_MOESM7_ESM.pdf]

nature portfolio

Corresponding author(s): S. Thomas Carmichael

Last updated by author(s): Oct 2, 2023

Reporting Summary

Nature Portfolio wishes to improve the reproducibility of the work that we publish. This form provides structure for consistency and transparency in reporting. For further information on Nature Portfolio policies, see our Editorial Policies and the Editorial Policy Checklist.

Statistics

For all statistical analyses, confirm that the following items are present in the figure legend, table legend, main text, or Methods section.

n/a	Confirmed	
[ ]	[x]	The exact sample size (*n*) for each experimental group/condition, given as a discrete number and unit of measurement
[ ]	[x]	A statement on whether measurements were taken from distinct samples or whether the same sample was measured repeatedly
[ ]	[x]	The statistical test(s) used AND whether they are one- or two-sided<br>*Only common tests should be described solely by name; describe more complex techniques in the Methods section.*
[x]	[ ]	A description of all covariates tested
[ ]	[x]	A description of any assumptions or corrections, such as tests of normality and adjustment for multiple comparisons
[ ]	[x]	A full description of the statistical parameters including central tendency (e.g. means) or other basic estimates (e.g. regression coefficient) AND variation (e.g. standard deviation) or associated estimates of uncertainty (e.g. confidence intervals)
[ ]	[x]	For null hypothesis testing, the test statistic (e.g. *F*, *t*, *r*) with confidence intervals, effect sizes, degrees of freedom and *P* value noted<br>*Give P values as exact values whenever suitable.*
[x]	[ ]	For Bayesian analysis, information on the choice of priors and Markov chain Monte Carlo settings
[x]	[ ]	For hierarchical and complex designs, identification of the appropriate level for tests and full reporting of outcomes
[x]	[ ]	Estimates of effect sizes (e.g. Cohen's *d*, Pearson's *r*), indicating how they were calculated

Our web collection on statistics for biologists contains articles on many of the points above.

Software and code

Policy information about availability of computer code

Data collection	ImageJ/FIJI (v. 2.0.0/1.53c); Microsoft Excel for Mac (v. 16.29); SerialCloner (v. 2.6.1); NIS Elements (v. 5.11.03); Imaris (v. 9.9); and NEBuilder were used in data collection.
Data analysis	Microsoft Excel for Mac (v. 16.29); GraphPad Prism (v. 8); R studio (v. 3.4.3); Gene Set Enrichment Analysis (v. 4.3.2); BioVenn; Benchling; and BioRender were used in data analysis and visualization.

For manuscripts utilizing custom algorithms or software that are central to the research but not yet described in published literature, software must be made available to editors and reviewers. We strongly encourage code deposition in a community repository (e.g. GitHub). See the Nature Portfolio guidelines for submitting code & software for further information.

Data

Policy information about availability of data

All manuscripts must include a data availability statement. This statement should provide the following information, where applicable:

- Accession codes, unique identifiers, or web links for publicly available datasets
- A description of any restrictions on data availability
- For clinical datasets or third party data, please ensure that the statement adheres to our policy

The following plasmids are available on Addgene: AAV-GfaABC1D-Cre-4x6T (196410), AAV-GfaABC1D-ERCreER-4x6T (196411), AAV-GfaABC1D-iCreV-4x6T (196412), AAV-GfaABC1D-DreO-4x6T (196413), AAV-GfaABC1D-lck-smFLAG-4x6T-WPRE (196414), AAV-GfaABC1D-lck-smMyc-4x6T-WPRE (196415), AAV-GfaABC1D-lck-smV5-4x6T-WPRE (196416), AAV-GfaABC1D-MCS--4x6T-WPRE (196417), AAV-CAG-flex-GFP-4x6T (196418), AAV-CAG-flex-lck-smV5-4x6T (196419), AAV-CAG-flex-

MCS--4x6T (196420), AAV-CAG-dDIO-lck-smMyc-4x6T (196421), AAV-CAG-dDIO-MCS--4x6T (196422), AAV-CAG-flex-lck-smV5 (196423). The Ribotag input and immunoprecipitated RNAseq datasets generated during the current study are available on GEO: GSE226366. Due to their large size, imaging datasets are available upon request; source data for imaging datasets are provided with this paper.

Human research participants

Policy information about studies involving human research participants and Sex and Gender in Research.

Reporting on sex and gender	*Use the terms sex (biological attribute) and gender (shaped by social and cultural circumstances) carefully in order to avoid confusing both terms. Indicate if findings apply to only one sex or gender; describe whether sex and gender were considered in study design whether sex and/or gender was determined based on self-reporting or assigned and methods used. Provide in the source data disaggregated sex and gender data where this information has been collected, and consent has been obtained for sharing of individual-level data; provide overall numbers in this Reporting Summary. Please state if this information has not been collected. Report sex- and gender-based analyses where performed, justify reasons for lack of sex- and gender-based analysis.*
Population characteristics	*Describe the covariate-relevant population characteristics of the human research participants (e.g. age, genotypic information, past and current diagnosis and treatment categories). If you filled out the behavioural & social sciences study design questions and have nothing to add here, write "See above."*
Recruitment	*Describe how participants were recruited. Outline any potential self-selection bias or other biases that may be present and how these are likely to impact results.*
Ethics oversight	*Identify the organization(s) that approved the study protocol.*

Note that full information on the approval of the study protocol must also be provided in the manuscript.

Field-specific reporting

Please select the one below that is the best fit for your research. If you are not sure, read the appropriate sections before making your selection.

- [x] Life sciences
- [ ] Behavioural & social sciences
- [ ] Ecological, evolutionary & environmental sciences

For a reference copy of the document with all sections, see nature.com/documents/nr-reporting-summary-flat.pdf

Life sciences study design

All studies must disclose on these points even when the disclosure is negative.

Sample size	Sample size was determined based on established practices in the field for assessing cell-type specificity of adeno-associated viruses using 3-5 animals per group (e.g. Deverman et al., Nat Biotech 2016; Chan et al., Nat Neuro 2017; Wang et al., Cell 2021; Lin et al., Nat Methods 2022).
Data exclusions	No data were excluded.
Replication	Experiments were independently performed at least 3 times, with the exception of negative control inducible Cre experiments, which were replicated twice (no tamoxifen for ER-Cre-ER induction, no light for iCreV induction). All replicates for each experiment had similar results.
Randomization	For transcriptomic experiments, male and female Ribotag+ mice 2-5 months were distributed across viral groups such that each group included both sexes and animals across the age range. For immunohistochemistry experiments, animals were distributed across viral groups such that each group included both sexes and animals from multiple cages.
Blinding	Identical protocols were used to process and analyze all samples. For direct comparison of the efficacy of different individual miR targeting sequences and cell-type specificity in different serotypes, experimenters were blinded during analysis. The nature of other comparisons prevented blinding: comparing different brain regions, systemic vs targeted viral expression, different reporters with different visual appearance, and comparing animals of different ages (due to the heavy presence of lipofuscin in 28m animals).

Reporting for specific materials, systems and methods

We require information from authors about some types of materials, experimental systems and methods used in many studies. Here, indicate whether each material, system or method listed is relevant to your study. If you are not sure if a list item applies to your research, read the appropriate section before selecting a response.

Materials & experimental systems

n/a	Involved in the study	
	X	Antibodies
	X	Eukaryotic cell lines
X		Palaeontology and archaeology
	X	Animals and other organisms
X		Clinical data
X		Dual use research of concern

Methods

n/a	Involved in the study	
X		ChIP-seq
X		Flow cytometry
X		MRI-based neuroimaging

Antibodies

Antibodies used	Primary antibodies used in this study: GFAP (1:1000, chicken, Rockland #200-901-D60); Aldh1l1 (1:400, rabbit, Abcam #Ab87117); Sox9 (1:1000, rabbit, Millipore Sigma #AB5535); NeuN (1:1000, chicken, Synaptic Systems #266 006); CD31 (1:100, rat, BD Biosciences #550274); V5 (1:400, human, Absolute Antibodies #AB00136-10.0); Myc (1:400, 488-labeled, Biotium #20436); FLAG (1:400, 543-labeled, Biotium #20433); HA (1:100, rat, Roche #11-867-423); GFP (1:500, nanobody, ChromoTek gba488-100). Secondary antibodies: 1:1000, Jackson ImmunoResearch, donkey anti-rabbit (488: 711-546-152; Cy3: 711-166-152; 647: 711-606-152), donkey anti-chicken (488: 703-546-155; Cy3: 703-166-155); donkey anti-rat (Cy3: 712-166-153; 647: 712-606-153); donkey anti-human (488: 709-546-159; 647: 709-606-149); 1:250, VioBlue-streptavidin (Miltenyi Biotec, 130-106-933).
Validation	All antibodies were commercially sourced and chosen for demonstrated specificity. Most have extensive references on BenchSci, an AI-assisted reagent selection tool:<br>Aldh1l1, Abcam Ab87117: 51 published references on BenchSci<br>Sox9, Millipore Sigma AB5535: 1360 published references on BenchSci<br>NeuN, Synaptic Systems 266 006: 19 published references on BenchSci<br>CD31, BD Bioscience 550274: 1549 published references on BenchSci<br>HA, Roche 11-867-423: 72 published references on BenchSci<br>GFP, ChromoTek gba488: 20 published references on BenchSci<br>GFAP, Rockland 200-901-D60: validated in our lab by comparing with other extensively published GFAP antibodies.<br>V5, Absolute Antibodies AB00136-10.0: recombinant antibody based on antibody first published in Randall et al., J Gen Virol 1987, with 150 citations.<br>Labeled Myc and FLAG antibodies: epitope tag antibodies validated in our lab by comparing staining in cells transduced with spaghetti monster viruses vs non-transduced control cells.

Eukaryotic cell lines

Policy information about cell lines and Sex and Gender in Research

Cell line source(s)	Adeno-associated viruses were produced in HEK293T cells, obtained from ATCC (CRL-3216).
Authentication	None.
Mycoplasma contamination	Not tested.
Commonly misidentified lines (See ICLAC register)	N/A

Animals and other research organisms

Policy information about studies involving animals; ARRIVE guidelines recommended for reporting animal research, and Sex and Gender in Research

Laboratory animals	This study used C57Bl/6J mice, ages postnatal day 1 to 28 months; the majority of experiments were conducted using 2-5 month old animals unless otherwise noted. Transgenic lines used: Ai14 (B6.Cg-Gt(ROSA)26Sortm14(CAG-dtTomato)Hze/J, RRID:IMSR_JAX:007914), RiboTag (B6N.129-Rpl22tm1.1Psam/J (RRID: IMSR_JAX: 011029), Aldh1l1-CreERT2 (B6/FVB-Tg(Aldh1l10-cre/ERT2)1Khakh/J, RRID:IMSR_JAX:029655).
Wild animals	The study did not involve wild animals.
Reporting on sex	Both male and female mice were used in this study; no sex-specific differences were observed.
Field-collected samples	The study did not involve field-collected samples.
Ethics oversight	The work using mice described in this study was conducted according to protocols approved by the University of California-Los Angeles Chancellor's Animal Research Committee.

Note that full information on the approval of the study protocol must also be provided in the manuscript.
